# Optimal Timing of Surgery Following Neoadjuvant Therapy for Surgical and Oncological Outcomes in Advanced Esophageal Squamous Cell Carcinoma: An Exploratory Analysis of JCOG1109

**DOI:** 10.1245/s10434-025-19050-6

**Published:** 2026-01-14

**Authors:** Ryosuke Kita, Ken Kato, Ryunosuke Machida, Yoshinori Ito, Hiroyuki Daiko, Hirofumi Kawakubo, Keita Sasaki, Haruhiko Fukuda, Yasue Kimura, Kohei Akiyoshi, Kazuo Koyanagi, Hiroki Hara, Keiko Minashi, Kenji Amagai, Tetsuya Abe, Satoru Matsuda, Takahiro Tsushima, Hiroya Takeuchi

**Affiliations:** 1 Japan Clinical Oncology Group Data Center/Operations Office, National Cancer Center Hospital, Tokyo, Japan; 2 Department of Surgery, Graduate School of Medicine, Kyoto University, Kyoto, Japan; 3 Department of Head and Neck, Esophageal Medical Oncology, National Cancer Center Hospital, Tokyo, Japan; 4 Department of Radiation Oncology, Showa University School of Medicine, Tokyo, Japan; 5 Department of Esophageal Surgery, National Cancer Center Hospital, Tokyo, Japan; 6 Department of Surgery, Keio University School of Medicine, Tokyo, Japan; 7 Department of Gastroenterological Surgery, National Hospital Organization (NHO) Kyushu Cancer Center, Fukuoka, Japan; 8 Department of Medical Oncology, Osaka City General Hospital, Osaka, Japan; 9 Department of Gastroenterological Surgery, Tokai University School of Medicine, Isehara, Japan; 10 Department of Gastroenterology, Saitama Cancer Center, Saitama, Japan; 11 Department of Gastroenterology, Chiba Cancer Center, Chiba, Japan; 12 Division of Gastroenterology, Ibaraki Prefectural Central Hospital and Cancer Center, Kasama, Japan; 13 Department of Gastroenterological Surgery, Aichi Cancer Center Hospital, Nagoya, Japan; 14 Division of Gastrointestinal Oncology, Shizuoka Cancer Center, Shizuoka, Japan; 15 Department of Surgery, Hamamatsu University School of Medicine, Hamamatsu, Japan

**Keywords:** Esophageal cancer, Squamous cell carcinoma, Neoadjuvant chemotherapy, Neoadjuvant chemoradiotherapy, Interval time

## Abstract

**Background:**

The optimal timing of surgery following neoadjuvant therapy in esophageal squamous cell carcinoma (ESCC) remains uncertain. This exploratory analysis aims to assess the impact of time to surgery (TTS) on perioperative and survival outcomes in patients with advanced ESCC enrolled in the phase III trial JCOG1109.

**Methods:**

Patients who underwent esophagectomy following neoadjuvant chemotherapy or chemoradiotherapy were included. Within each treatment arm, patients were categorized into four TTS subgroups according to cohort quartiles. Perioperative complications, overall survival (OS), and progression-free survival (PFS) were evaluated.

**Results:**

The median TTS was 35 (range, 16–81), 38 (17–109), and 41 (14–98) days for the cisplatin plus fluorouracil (CF), docetaxel, cisplatin plus fluorouracil, and CF with radiotherapy (CF-RT) arms, respectively. Baseline characteristics were comparable across the TTS subgroups. Operative time and overall complication rates showed no significant differences. In the CF-RT arm, a longer TTS was associated with increased blood loss (200, 210, 300, and 370 mL) and a trend toward a higher anastomotic leakage (6%, 7%, 14%, and 18%), as well as a trend toward poorer PFS and OS, particularly at more prolonged intervals. However, across all treatment arms, OS and PFS did not differ among the TTS subgroups, with multivariable analysis revealing no consistent associations between TTS and survival.

**Conclusions:**

Time to surgery following neoadjuvant therapy did not significantly influence prognosis in advanced ESCC. Nonetheless, prolonged intervals after chemoradiotherapy may increase surgical complexity and leakage risk, in addition to elevating the potential for adverse prognostic effects associated with excessive delays.

**Supplementary Information:**

The online version contains supplementary material available at 10.1245/s10434-025-19050-6.

Globally, esophageal cancer is the sixth-leading cause of cancer-related mortality accounting for 5.6% of all cancer-related deaths.^1–3^ Multimodal treatment strategies, including surgery, chemotherapy, and radiotherapy, have been developed for improving survival; however, regional practices vary according to tumor biology. Adenocarcinoma predominates in Western countries, where neoadjuvant chemoradiotherapy (CRT) is the standard approach, whereas esophageal squamous cell carcinoma (ESCC) is more common in East Asia, where chemotherapy-based regimens are widely employed.^4–6^

Despite these multimodal therapy advancements, the optimal timing of surgery following neoadjuvant treatment remains controversial. Previous studies have reported inconsistent findings regarding the association between time to surgery (TTS) and outcomes, particularly in CRT cohorts.^7–16^ The NeoRes II trial, primarily conducted in patients with adenocarcinoma, revealed no benefit from delaying surgery beyond 10–12 weeks following CRT, suggesting a trend toward worse survival.^17^ Data addressing the impact of TTS following neoadjuvant chemotherapy alone remain scarce.

JCOG1109, a randomized phase III trial, compared the following three neoadjuvant strategies for advanced ESCC: cisplatin and 5-fluorouracil (CF); docetaxel, cisplatin, and 5-fluorouracil (DCF); and cisplatin, 5-fluorouracil, and radiation therapy (CF-RT). The trial demonstrated the superiority of DCF over CF in improving overall survival (OS), resulting in its adoption as the current standard in Japan.^18^ Notably, this dataset facilitates evaluating the prognostic significance of surgical timing following chemotherapy and CRT.

This study aimed to evaluate the impact of TTS following neoadjuvant therapy on perioperative morbidity and long-term survival in patients with advanced ESCC, using data from JCOG1109.

## Methods

### Patients

Details of the eligibility criteria and study design for JCOG1109, a multicenter phase III randomized trial evaluating neoadjuvant treatments for esophageal cancer, have been previously described.^19^ The following were the key eligibility criteria: (1) histologically confirmed squamous cell carcinoma, adenosquamous carcinoma, or basaloid cell carcinoma of the thoracic esophagus; (2) clinical stage IB, II, or III, excluding T4 according to the seventh UICC TNM classification; (3) aged 20–75 years; and 4) Eastern Cooperative Oncology Group (ECOG) performance status 0–1. Patients were randomly allocated (1:1:1) to one of three neoadjuvant treatment arms as follows: CF, DCF, or CF-RT. This exploratory analysis encompassed eligible patients from JCOG1109 who underwent esophagectomy following receiving neoadjuvant therapy. All clinical data were obtained from the JCOG1109 database. Written informed consent was obtained from all patients, including consent for secondary data use. JCOG1109 is registered with the Japan Registry of Clinical Trials (jRCTs031180202).

### Definition of TTS and Subgrouping

Time to surgery was defined as the duration from the last day of neoadjuvant therapy to the date of surgery. Patients were stratified into four subgroups within each treatment arm on the basis of TTS distribution quantiles in the overall cohort.

### Neoadjuvant Treatments

In the CF arm, patients received two cycles of neoadjuvant CF (cisplatin 80 mg/m^2^/day on day 1 and 5-FU 800 mg/m^2^/day on days 1–5) at 3-week intervals. In the DCF arm, three cycles of neoadjuvant DCF (docetaxel 70 mg/m^2^/day and cisplatin 70 mg/m^2^/day on day 1, with 5-FU 750 mg/m^2^/day on days 1–5) were administered at 3-week intervals. In the CF-RT arm, neoadjuvant CRT was delivered with a total radiation dose of 41.4 Gy over 5 weeks (23 fractions), combined with two cycles of CF (cisplatin 75 mg/m^2^/day on day 1 and 5-FU 1,000 mg/m^2^/day on days 1–4) at 4-week intervals. The radiation fields covered the primary tumor with a 2-cm margin and regional lymph nodes according to tumor location. Surgery, either subtotal or total thoracic esophagectomy with regional lymphadenectomy via right thoracotomy, was performed within 56 days following neoadjuvant treatment. Reconstruction methods were not required and were left to the discretion of the surgeon, with gastric tube reconstruction and cervical anastomosis being the standard approach.

### Outcomes

Short-term outcomes encompassed intraoperative findings (number of dissected lymph nodes, proportion of R0 resection, operative time, and blood loss) and postoperative outcomes (pathological response rate assessed according to the Japanese Classification of Esophageal Cancer, 11th edition, and postoperative complications classified using the Common Terminology Criteria for Adverse Events version 4.0).^20^ Long-term outcomes consisted of OS, defined as the time from randomization to death from any cause, and progression-free survival (PFS), defined as the time from randomization to the first evidence of disease progression or death from any cause. Disease progression noted during neoadjuvant therapy was not considered a PFS event if followed by R0 or R1 resection; however, in cases of R2 resection, radiologically confirmed progression postoperatively was considered a PFS event.

### Statistical Analysis

Baseline characteristics across the TTS subgroups were compared using the Kruskal–Wallis and chi-square tests for continuous and categorical variables, respectively. Trends in perioperative outcomes across the TTS subgroups within each treatment arm were analyzed using the Jonckheere–Terpstra and Cochran–Armitage trend tests for continuous and categorical variables, respectively. Long-term outcomes, including OS and PFS, were estimated using the Kaplan–Meier method. Hazard ratios (HR) and corresponding 95% confidence intervals (CI) for the TTS subgroups were calculated by using the Cox proportional hazards model. Multivariable analysis was adjusted for age, sex, ECOG performance status, neoadjuvant therapy completion, and clinical stage. All *p*-values were two-sided, and statistical analyses were performed using SAS version 9.4 (SAS Institute, Cary, NC).

## Results

### Patient Characteristics and TTS Distribution

From December 2012 to July 2018, 601 patients were enrolled in JCOG1109 and randomly allocated to one of three neoadjuvant treatment groups: CF (*n* = 199), DCF (*n* = 202), and CF-RT (*n* = 200). Of the patients, 546 proceeded to esophagectomy following neoadjuvant therapy (CF, *n* = 185; DCF, *n* = 183; CF-RT, *n* = 178). The flowchart of patient enrollment, exclusions, and surgical cases is depicted in Supplementary Fig. 1 (Supplemental Digital Content 1). In the overall cohort, the median age was 65 (range, 30–75) years, with almost all patients having squamous cell carcinoma histology. In 58.6% of patients, the tumor was located in the middle thoracic esophagus. The completion proportion of neoadjuvant therapy was 92.1%, and 93.8% of patients underwent surgery within 56 days following neoadjuvant therapy completion, in accordance with protocol specifications. The overall median TTS was 38 (range, 14–109) days, with median TTS of 35 (range, 16–81), 38 (range, 17–109), and 41 (range, 14–98) days in the CF, DCF, and CF-RT arms, respectively (Table 1). To maximize the statistical power for detecting potential trends, the main analysis adopted a quantile-based classification of TTS, ensuring balanced patient numbers across the subgroups within each treatment arm. Based on TTS quantiles of the overall cohort, patients were stratified into the following four groups: Q1 (<32 days), Q2 (32–37 days), Q3 (38–46 days), and Q4 (≥47 days). The following was the distribution of patients across these subgroups 64, 50, 43, and 28 patients in Q1–Q4 of the CF arm; 46, 38, 50, and 49 in the DCF arm; and 17, 41, 59, and 61 in the CF-RT arm. Generally, the demographic and clinical characteristics of the patients were comparable across all TTS subgroups within each treatment arm, except for age, body mass index, and neoadjuvant treatment completion proportions in the DCF arm (Table 2).

**Table 1:** Distribution of interval time between neoadjuvant treatment and surgery

	CF arm (*n* = 185)	DCF arm (*n* = 183)	CF-RT arm (*n* = 178)	Toal (*n* = 546)
Interval time, days				
Median	35	38	41	38
Range	16–81	17–109	14–98	14–109
25–75%	30–42	31–48	35–49	32–47
Stratified by absolute values				
Early (<28 day)	22	20	9	51
Middle (28–56 day)	152	150	155	457
Late (>56 day)	11	13	14	38
Stratified by quantile values (pooled population)				
<25% (32 day)	64	46	17	127
≥25% (32 day), <50% (38 day)	50	38	41	129
≥50% (38 day), <75% (47 day)	43	50	59	152
≥75% (47 day)	28	49	61	138
Stratified by quantile values (each arm)				
<25%	43	38	32	113
≥25%, <50%	48	46	49	143
≥50%, <75%	46	51	49	146
≥75%	48	48	48	144

**Table 2:** **a** Patient characteristics in CF arm. **b** Patient characteristics in DCF arm. **c** Patient characteristics in CF-RT arm

	Q1 *n* = 64	Q2 *n* = 50	Q3 *n* = 43	Q4 *n* = 28	*p*
Age, years	64 (46–75)	65 (48–75)	65 (49–74)	64 (38–74)	0.935
<65	33 (52)	22 (44)	20 (47)	16 (57)	0.679
≥65	31 (48)	28 (56)	23 (53)	12 (43)	
Gender					
Male	60 (94)	43 (86)	38 (88)	24 (86)	0.515
Female	4 (6)	7 (14)	5 (12)	4 (14)	
BMI, kg/m^2^	21.8 (14.7–27.5)	22.4 (16.4–27.6)	21.8 (15.9–27.8)	22.8 (16.5–26.1)	0.401
ECOG PS					
0	55 (86)	44 (88)	34 (79)	22 (79)	0.545
1	9 (14)	6 (12)	9 (21)	6 (21)	
Histology					
SCC	64 (100)	49 (98)	43 (100)	27 (96)	0.364
Others	0 (0)	1 (2)	0 (0)	1 (4)	
Tumor location					
Ut	8 (13)	9 (18)	2 (5)	7 (25)	0.232
Mt	37 (58)	27 (54)	31 (72)	15 (54)	
Lt	19 (30)	14 (28)	10 (23)	6 (21)	
Clinical stage (UICC-TNM, 7th edition)					
IB	6 (9)	2 (4)	4 (9)	2 (7)	0.207
II	13 (20)	19 (38)	14 (33)	13 (46)	
III	45 (70)	29 (58)	25 (58)	13 (46)	
Neoadjuvant treatment completion					0.165
Yes	60 (94)	47 (94)	41 (95)	23 (82)	
No	4 (6)	3 (6)	2 (5)	5 (18)	
Adverse event of neoadjuvant treatment					
≤ Grade 2	48 (75)	33 (66)	26 (61)	17 (61)	0.361
> Grade 2	16 (25)	17 (34)	17 (40)	11 (39)	

	Q1 *n* = 46	Q2 *n* = 38	Q3 *n* = 50	Q4 *n* = 49	*p*
Age, years	61 (42–75)	64 (43–75)	66 (41–75)	66 (45–75)	0.028
<65	31 (67)	21 (55)	22 (44)	19 (39)	0.028
≥65	15 (33)	17 (45)	28 (56)	30 (61)	
Gender					0.840
Male	40 (87)	33 (87)	46 (92)	43 (88)	
Female	6 (13)	5 (13)	4 (8)	6 (12)	
BMI, kg/m^2^	21.5 (15.8–29.6)	22.0 (16.6–29.0)	22.7 (16.4–30.2)	21.0 (15.7–27.7)	0.039
ECOG PS					0.183
0	40 (87)	34 (90)	47 (94)	39 (80)	
1	6 (13)	4 (11)	3 (6)	10 (20)	
Histology					0.143
SCC	46 (100)	38 (100)	49 (98)	46 (94)	
Others	0 (0)	0 (0)	1 (2)	3 (6)	
Tumor location					0.528
Ut	3 (7)	7 (18)	8 (16)	4 (8)	
Mt	28 (61)	20 (53)	28 (56)	26 (53)	
Lt	15 (33)	11 (29)	14 (28)	19 (39)	
Clinical stage (UICC-TNM, 7th edition)	0.947				
IB	4 (9)	2 (5)	6 (12)	5 (10)	
II	12 (26)	12 (32)	13 (26)	15 (31)	
III	30 (65)	24 (63)	31 (62)	29 (59)	
Neoadjuvant treatment completion					0.037
Yes	44 (96)	31 (82)	48 (96)	41 (84)	
No	2 (4)	7 (18)	2 (4)	8 (16)	
Adverse event of neoadjuvant treatment					0.819
≤ Grade 2	3 (7)	2 (5)	5 (10)	3 (6)	
> Grade 2	43 (94)	36 (95)	45 (90)	46 (94)	

	Q1 *n* = 17	Q2 *n* = 41	Q3 *n* = 59	Q4 *n* = 61	*p*
Age, years	65 (53-72)	63 (30-74)	66 (44-75)	65 (45-74)	0.207
<65	8 (47)	21 (51)	26 (44)	28 (46)	0.915
≥65	9 (53)	20 (49)	33 (56)	33 (54)	
Gender					0.840
Male	16 (94)	33 (80)	50 (85)	54 (89)	0.501
Female	1 (6)	8 (20)	9 (15)	7 (11)	
BMI, kg/m^2^	21.3 (16.9–27.2)	22.7 (16.9–27.8)	22.1 (15.6–31.1)	21.0 (15.4–30.8)	0.297
ECOG PS					0.461
0	16 (94)	36 (88)	53 (90)	50 (82)	
1	1 (6)	5 (12)	6 (10)	11 (18)	
Histology					0.308
SCC	17 (100)	41 (100)	56 (95)	60 (98)	
Others	0 (0)	0 (0)	3 (5)	1 (2)	
Tumor location					0.253
Ut	1 (6)	1 (2)	5 (9)	9 (15)	
Mt	11 (65)	26 (63)	32 (54)	39 (64)	
Lt	5 (30)	14 (34)	22 (37)	13 (21)	
Clinical stage (UICC-TNM, 7th edition)					0.309
IB	0 (0)	6 (15)	6 (10)	7 (12)	
II	7 (41)	11 (27)	19 (32)	11 (18)	
III	10 (59)	24 (59)	34 (58)	43 (71)	
Neoadjuvant treatment completion					0.466
Yes	17 (100)	38 (93)	57 (97)	56 (92)	
No	0 (0)	3 (7)	2 (3)	5 (8)	
Adverse event of neoadjuvant treatment					0.867
≤ Grade 2	5 (29)	15 (37)	17 (29)	19 (31)	
> Grade 2	12 (91)	26 (63)	42 (71)	42 (69)	

Data are median (range) or no. (%). Percentages might not add up to 100 because of rounding. Adverse events related to neoadjuvant treatment were assessed by CTCAE ver.4.0. *CF* cisplatin and 5-fluorouracil; *DCF* docetaxel, cisplatin, and 5-fluorouracil; *CF-RT* cisplatin, 5-fluorouracil, and radiation therapy; *BMI* body mass index; *TNM* Tumor Node Metastasis; *UICC* International Union for Cancer Control

### Perioperative Outcomes

The operative procedures and perioperative outcomes, including surgical approach, extent of lymph node dissection, operative time, residual tumor status, and length of postoperative hospital stay, exhibited no significant differences across the TTS subgroups within each treatment arm (Table 3). In the CF-RT arm, a gradual increase in intraoperative blood loss was noted across the TTS subgroups from Q1 to Q4 (Q1: 200 mL, Q2: 210 mL, Q3: 300 mL, Q4: 370 mL; *p* = 0.010). Conversely, the number of harvested lymph nodes progressively decreased with longer TTS from 54 in Q1 to 42 in Q4 (*p* = 0.010). Although the overall incidence of postoperative complications did not significantly differ between the TTS subgroups, the CF-RT arm demonstrated a trend toward a higher anastomotic leakage proportion with longer TTS (Q1: 5.9%, Q2: 7.3%, Q3: 13.6%, and Q4: 18.0%; *p* = 0.073). Regarding pathological response, in the CF-RT arm, the proportion of patients achieving Grade 2 (≥two-thirds tumor necrotic/fibrotic) and Grade 3 (complete absence of viable tumor cells) progressively decreased from Q1 to Q4 (Q1: 88.2%, Q2: 92.7%, Q3: 78%, and Q4: 65%; *p* = 0.001).

**Table 3:** **a** Short-term outcomes in CF arm. **b** Short-term outcomes in DCF arm. **c** Short-term outcomes in CF-RT arm

	Q1 *n* = 64	Q2 *n* = 50	Q3 *n* = 43	Q4 *n* = 28	*p*
Thoracic approach					0.847
Thoracotomy	29 (45)	30 (60)	21 (49)	12 (43)	
Thoracoscopic surgery	35 (55)	20 (40)	22 (51)	16 (57)	
Abdominal approach					0.315
Laparotomy	33 (52)	32 (65)	21 (49)	12 (43)	
Laparoscopic surgery	30 (48)	17 (35)	22 (51)	16 (57)	
LN dissection					0.962
< D2	3 (5)	3 (6)	5 (12)	0 (0)	
≥ D2	60 (95)	46 (94)	38 (88)	28 (100)	
LN counts	57 (24–122)	55 (25–103)	66 (27–125)	62 (24–112)	0.215
Operation time, min	496 (107–770)	490 (359–724)	489 (326–743)	513 (303–662)	0.991
Blood loss, ml	250 (15–1156)	346 (25–1568)	312 (49–1246)	288 (17–1767)	0.092
Residual tumor					0.134
R0	59 (92)	48 (96)	41 (95)	28 (100)	
R1-2	5 (8)	2 (4)	2 (5)	0 (0)	
Postoperative stay, days	21 (10–93)	20 (12–88)	20 (12–165)	18 (11–50)	0.120
Pathological stage					0.090
< IIIA	23 (37)	24 (49)	24 (56)	14 (50)	
≥ IIIA	40 (64)	25 (51)	19 (44)	14 (50)	
Pathological response					0.314
< Grade 2	50 (79)	38 (78)	37 (86)	24 (86)	
≥ Grade 2	13 (21)	11 (22)	6 (14)	4 (14)	
Postoperative complications					
Any	39 (61)	28 (56)	23 (54)	15 (54)	0.417
Anastomotic leakage	7 (11)	6 (12)	5 (12)	1 (4)	0.417

	Q1 *n* = 46	Q2 *n* = 38	Q3 *n* = 50	Q4 *n* = 49	*p*
Thoracic approach					0.587
Thoracotomy	26 (57)	16 (42)	24 (48)	24 (49)	
Thoracoscopic surgery	20 (44)	22 (58)	26 (52)	25 (51)	
Abdominal approach					0.055
Laparotomy	33 (72)	19 (50)	20 (41)	26 (54)	
Laparoscopic surgery	13 (28)	19 (50)	29 (59)	22 (46)	
LN dissection					0.003
< D2	1 (2)	0 (0)	1 (2)	8 (17)	
≥ D2	45 (98)	38 (100)	48 (98)	40 (83)	
LN counts	66 (19–122)	58 (25–143)	58 (27–110)	56 (23–93)	0.008
Operation time, min	506 (282–758)	466 (274–667)	483 (222–712)	478 (285–813)	0.806
Blood loss, ml	285 (13–974)	189 (10–1050)	296 (20–1,050)	341 (37–3,490)	0.288
Residual tumor					0.181
R0	46 (100)	36 (95)	49 (98)	46 (94)	
R1-2	0 (0)	2 (5)	1 (2)	3 (6)	
Postoperative stay, days	20 (10–96)	17 (10–96)	17 (10–100)	21 (11–85)	0.976
Pathological stage					0.950
< IIIA	31 (67)	24 (63)	30 (61)	33 (69)	
≥ IIIA	15 (33)	14 (37)	19 (39)	15 (31)	
Pathological response					0.880
< Grade 2	20 (44)	18 (47)	22 (45)	22 (46)	
≥ Grade 2	26 (57)	20 (53)	27 (55)	26 (54)	
Postoperative complications					
Any	19 (41)	21 (55)	18 (36)	22 (45)	0.209
Anastomotic leakage	4 (9)	1 (3)	4 (8)	7 (14)	0.241

	Q1 *n* = 17	Q2 *n* = 41	Q3 *n* = 59	Q4 *n* = 61	*p*
Thoracic approach					0.627
Thoracotomy	9 (53)	19 (46)	30 (51)	33 (54)	
Thoracoscopic surgery	8 (47)	22 (54)	29 (49)	28 (46)	
Abdominal approach					0.159
Laparotomy	10 (59)	25 (61)	30 (51)	28 (47)	
Laparoscopic surgery	7 (41)	16 (39)	29 (49)	32 (53)	
LN dissection					0.487
< D2	0 (0.0)	0 (0.0)	3 (5)	1 (2)	
≥ D2	17 (100)	41 (100)	56 (95)	59 (98)	
LN counts	54 (21–97)	53 (22–95)	49 (14–148)	42 (11–98)	0.010
Operation time, min	495 (271–720)	485 (325–672)	501 (249–780)	491 (262–716)	0.754
Blood loss, ml	200 (60–550)	210 (8–900)	300 (50–1599)	370 (13–1831)	0.010
Residual tumor					0.181
R0	17 (100)	41 (100)	58 (98)	59 (97)	
R1-2	0 (0)	0 (0)	1 (2)	2 (3)	
Postoperative stay, days	21 (13–121)	19 (11–102)	21 (11–104)	17 (7–109)	0.301
Pathological Stage					0.022
< IIIA	15 (88)	35 (85)	48 (81)	41 (68)	
≥ IIIA	2 (12)	6 (15)	11 (19)	19 (32)	
Pathological response					0.001
< Grade 2	2 (12)	3 (7)	13 (22)	21 (35)	
≥ Grade 2	15 (88)	38 (93)	46 (78)	39 (65)	
Postoperative complications					
Any	4 (24)	19 (46)	31 (53)	31 (51)	0.100
Anastomotic leakage	1 (6)	3 (7)	8 (14)	11 (18)	0.073

Data are median (range) or no. (%). Percentages might not add up to 100 because of rounding. *CF* cisplatin and 5-fluorouracil; *DCF* docetaxel, cisplatin, and 5-fluorouracil; *CF-RT* cisplatin, 5-fluorouracil, and radiation therapy

### Survival Outcomes

**Fig. 1:**
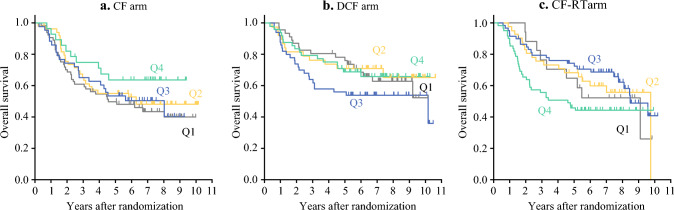
Overall survival in the TTS subgroups. Kaplan–Meier curves for overall survival according to quartiles (Q1–Q4) in each treatment arm, with patients stratified into four subgroups within each treatment arm on the basis of TTS distribution quantiles in the overall cohort. **a** CF arm, **b** DCF arm, and **c** CF-RT arm. *CF* cisplatin plus fluorouracil; *DCF* docetaxel, cisplatin plus fluorouracil; *CF-RT* CF with radiotherapy

**Fig. 2:**
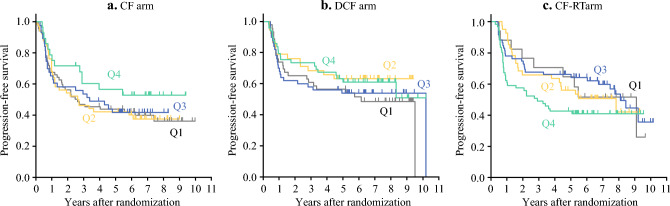
Progression-free survival in the TTS subgroups. Kaplan–Meier curves for progression-free survival according to quartiles (Q1–Q4) in each treatment arm. **a** CF arm, **b** DCF arm, and **c** CF-RT arm. *CF* cisplatin plus fluorouracil; *DCF* docetaxel, cisplatin plus fluorouracil; *CF-RT* CF with radiotherapy

**Fig. 3:**
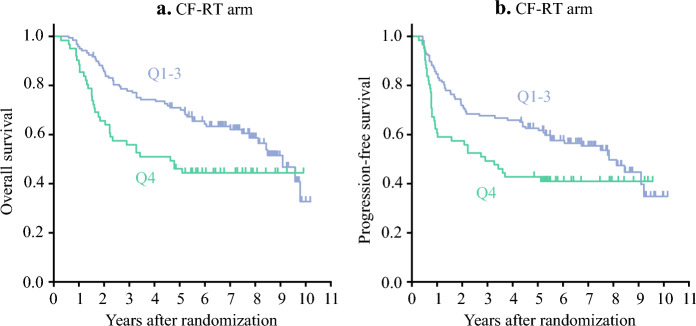
Overall survival and progression-free survival comparing the Q1-Q3 and Q4 TTS subgroups in the CF-RT arm. Kaplan–Meier curves for overall survival and progression-free survival in the CF-RT arm, comparing patients in the Q1–Q3 TTS subgroups with those in the Q4 subgroup. *CF-RT* cisplatin plus fluorouracil with radiotherapy

The median follow-up duration was 67.5 months, during which 284 PFS events and 247 deaths were observed. The 5-year OS across the TTS subgroups from Q1 to Q4 were 49.8%, 55.1%, 53.5%, and 63.6% in the CF arm (*p* = 0.386), 78.2%, 71.1%, 56%, and 68.9% in the DCF arm (*p* = 0.294); and 64.7%, 68.3%, 74.3%, and 45.9% in the CF-RT arm, respectively (*p* = 0.083) (Fig. 1). Similarly, the 5-year PFS from Q1 to Q4 across the TTS subgroups were 43.7%, 42%, 41.5%, and 56.4% in the CF arm (*p* = 0.587); 56.5%, 63.2%, 53.9%, and 61.1% in the DCF arm (*p* = 0.555); and 64.7%, 56.1%, 66.1%, and 42.6% in the CF-RT arm, respectively (*p* = 0.228; Fig. 2). The TTS subgroups within any treatment arm revealed no significant differences in OS or PFS. However, in the CF-RT arm, a comparison between Q1 to Q3 and Q4 showed a tendency toward worse PFS and OS in the Q4 subgroup (Fig. 3). Multivariable analysis adjusted for age, sex, ECOG performance status, neoadjuvant therapy completion, and clinical stage demonstrated no significant differences in HR across the TTS subgroups (Table 4; Supplementary Table 1). Moreover, sensitivity analyses using quantile-based stratification within each treatment arm and absolute value-based stratification, alternative TTS classifications, consistently indicated that TTS variations were not associated with prognostic differences in any treatment arms (Supplementary Tables 2 and 3; Supplementary Figs. 2 and 3; Supplemental Digital Content 1).

**Table 4:** Univariable and multivariable analysis for overall survival

		CF arm			DCF arm			CF-RT arm		
		Univariable	Multivariable		Univariable	Multivariable		Univariable	Multivariable	
		HR (95% CI)	HR (95% CI)	*p*	HR (95% CI)	HR (95% CI)	*p*	HR (95% CI)	HR (95% CI)	*p*
Age (years)	<65	1	1		1	1		1	1	
	≥65	1.152 (0.766-1.732)	1.139 (0.744-1.742)	0.549	0.971 (0.605-1.558)	0.801 (0.489-1.313)	0.380	1.618 (1.042-2.513)	1.613 (1.030-2.526)	0.037
Gender	Male	1	1		1	1		1	1	
	Female	0.460 (0.201-1.054)	0.447 (0.193-1.038)	0.061	0.413 (0.151-1.135)	0.437 (0.158-1.208)	0.111	0.770 (0.397-1.491)	0.693 (0.348-1.383)	0.299
ECOG PS	0	1	1		1	1		1	1	
	1	1.127 (0.648-1.959)	1.007 (0.569-1.784)	0.980	0.273 (0.086-0.869)	0.246 (0.075-0.803)	0.020	0.966 (0.499-1.869)	0.865 (0.443-1.687)	0.670
Neoadjuvant treatment completion	No	1	1		1	1		1	1	
	Yes	0.529 (0.266-1.054)	0.441 (0.216-0.901)	0.025	0.983 (0.449-2.148)	0.977 (0.437-2.186)	0.955	0.350 (0.161-0.763)	0.433 (0.189-0.988)	0.047
cStage	IB	1	1		1	1		1	1	
	II	0.611 (0.258-1.446)	0.664 (0.273-1.613)	0.366	7.629 (1.020-57.028)	8.530 (1.137-63.977)	0.037	0.668 (0.305-1.460)	0.724 (0.326-1.608)	0.428
	III	1.399 (0.642-3.051)	1.448 (0.647-3.238)	0.368	9.272 (1.279-67.200)	11.231 (1.542-81.823)	0.017	1.181 (0.603-2.311)	1.235 (0.625-2.438)	0.544
Interval time	Q1	1	1		1	1		1	1	
	Q2	0.822 (0.494-1.370)	0.998 (0.587-1.697)	0.994	0.892 (0.426-1.869)	0.899 (0.425-1.901)	0.781	0.907 (0.407-2.021)	0.850 (0.371-1.947)	0.701
	Q3	0.874 (0.514-1.486)	1.037 (0.602-1.786)	0.896	1.532 (0.822-2.857)	1.556 (0.820-2.952)	0.176	0.783 (0.364-1.685)	0.745 (0.343-1.618)	0.457
	Q4	0.544 (0.270-1.096)	0.643 (0.312-1.328)	0.233	0.941 (0.475-1.863)	1.211 (0.589-2.492)	0.602	1.488 (0.712-3.108)	1.274 (0.598-2.716)	0.531

*HR* Hazard ratio, *CI* Confidence interval

## Discussion

This exploratory analysis of JCOG1109 evaluated the impact of surgical timing following neoadjuvant therapy on short- and long-term outcomes in patients with advanced ESCC. Variations in TTS did not significantly impact postoperative complications, PFS, or OS irrespective of the treatment regimen. However, a slight tendency toward increased intraoperative blood loss and a higher anastomotic leakage incidence was noted with longer TTS in the neoadjuvant CRT arm, and patients with the most prolonged intervals also showed a trend toward poorer PFS and OS.

Most previous studies on the impact of the interval between neoadjuvant therapy and surgery have been retrospective, where longer TTS is frequently associated with factors, including treatment toxicity or delayed recovery.^14,21–23^ However, patient-related factors or heterogeneity in patient selection and treatment have not been fully accounted for in these studies, thereby limiting their ability to draw definitive conclusions. The NeoRes II trial, a randomized controlled trial, investigated the effects of prolonged TTS following neoadjuvant CRT in patients with resectable locally advanced esophageal cancer. The trial reported a trend toward worse survival, suggesting caution in delaying surgery beyond 6 weeks.^17^ Similarly, the GRECCAR-6 trial on rectal cancer revealed that extended TTS did not improve survival outcomes and was associated with higher morbidity and more difficult surgery.^24,25^ To the best of our knowledge, this is the first study to utilize data from a randomized trial to evaluate homogeneous cohorts of patients with ESCC. Furthermore, although most prior studies have focused on TTS following neoadjuvant CRT, this study uniquely investigated TTS following neoadjuvant chemotherapy, demonstrating that surgical timing did not affect postoperative complications or prognosis.

In the CF-RT arm, longer TTS was associated with increased intraoperative blood loss, a higher anastomotic leakage incidence, and a reduced number of harvested lymph nodes, as well as a tendency toward poorer PFS and OS, particularly when the interval approached the time frame examined in the NeoRes II trial. These perioperative and survival patterns are consistent with those observed in that study in patients with ESCC. Although radiation-induced damage to lymph node structures may have contributed to the reduced number of harvested lymph nodes, the impact of increased therapy-induced fibrosis and local inflammation due to prolonged TTS is probably more significant. Previous animal studies have demonstrated that radiation-treated esophagi exhibit significant changes in tissue layers, including decreased collagen fiber density in the lamina propria and collagen fiber infiltration into the muscularis mucosae over time.^26,27^ Notably, similar alterations are noted in muscle tissue, where collagen fiber deposition increases, and the expression of fibrosis markers also increases over time following irradiation.^28^ These findings suggest that radiation therapy causes esophageal tissue thickening and continuous fibrosis development over time. Such changes can complicate the surgical procedure and impair tissue viability, thereby affecting surgical complexity and postoperative outcomes, potentially contributing to the observed increases in blood loss and anastomotic leakage.

Moreover, in the CF-RT arm, a longer TTS was associated with a lower proportion of patients achieving pathological response of grade ≥2. Although previous studies have suggested that prolonged TTS does not adversely affect, or may even improve, pathological response rates, our study showed that prolonged TTS was associated with reduced pathological response.^7,16,29^ The underlying mechanisms remain unclear, and further investigations, including larger-scale studies and molecular biological analyses, are warranted. The lower pathological response rate in patients with extended TTS may have contributed to a higher proportion of patients with pathological stage III or higher in the CF-RT arm. This relative increase in advanced tumors may have further complicated the surgical procedure, potentially leading to the observed increased intraoperative blood loss and radiation-induced fibrosis. Nevertheless, no significant differences in OS or PFS were noted, suggesting that sufficient local control was achieved through surgery, irrespective of the timing of surgery.

This study had several limitations. First, the protocol-specified requirement for surgery within 56 days of completing neoadjuvant therapy hindered the evaluation of the study participants. With a protocol adherence proportion of 90%, only a small number of patients had TTS >56 days, limiting the ability to completely evaluate the influence of TTS beyond this threshold. However, within a 56-day window, TTS variations did not appear to influence postoperative outcomes or prognosis. Second, although TTS duration may be more influenced by institutional factors than patient characteristics, these institutional variations were not considered in our analysis. Third, the molecular mechanisms underlying the effect of TTS on tissue behavior and response to treatment remain unclear, warranting further research into the biological implications of prolonged TTS on tissue characteristics. Finally, as this is an *ad hoc* analysis, prospective clinical studies are warranted to validate these findings.

## Conclusions

The timing of surgery following neoadjuvant therapy did not influence prognosis in patients with advanced ESCC withing 56-day interval, regardless of the neoadjuvant therapy regimen. However, earlier surgery following neoadjuvant CRT, as opposed to neoadjuvant chemotherapy, may help to mitigate surgical complexity and anastomotic leakage-associated risks, and could contribute to avoiding potential adverse prognostic impacts associated with delayed surgery.

## Supplementary Information

Below is the link to the electronic supplementary material.

Supplementary file1 (DOCX 431 KB)
