# Supplementary material for: Optimal Timing of Surgery Following Neoadjuvant Therapy for Surgical and Oncological Outcomes in Advanced Esophageal Squamous Cell Carcinoma: An Exploratory Analysis of JCOG1109
Source: Ann Surg Oncol. 2026 Jan 14;33(6):5327–38. doi: 10.1245/s10434-025-19050-6 (PMC13179169; doi:10.1245/s10434-025-19050-6)
Supplement: Supplementary file 1 — Supplementary file1 (DOCX 431 KB) [file 10434_2025_19050_MOESM1_ESM.docx]

**Supplementary Figure 1. Patient flow diagram**

Flow diagram of patient enrollment, treatment, and surgery.

CF = cisplatin plus fluorouracil, DCF = docetaxel, cisplatin plus fluorouracil, CF-RT = CF with radiotherapy.

**Supplementary Figure 2. Overall survival in the TTS subgroups with quantile-based stratification**

Kaplan–Meier curves for overall survival with quantile-based stratification within each treatment arm. (a) CF arm, (b) DCF arm, and (c) CF-RT arm.
CF = cisplatin plus fluorouracil, DCF = docetaxel, cisplatin plus fluorouracil, CF-RT = CF with radiotherapy.

**Supplementary Figure 3. Overall survival in the TTS subgroups with absolute value-based stratification**

Kaplan–Meier curves for overall survival with absolute value-based stratification within each treatment arm. (a) CF arm, (b) DCF arm, and (c) CF-RT arm.
CF = cisplatin plus fluorouracil, DCF = docetaxel, cisplatin plus fluorouracil, CF-RT = CF with radiotherapy.


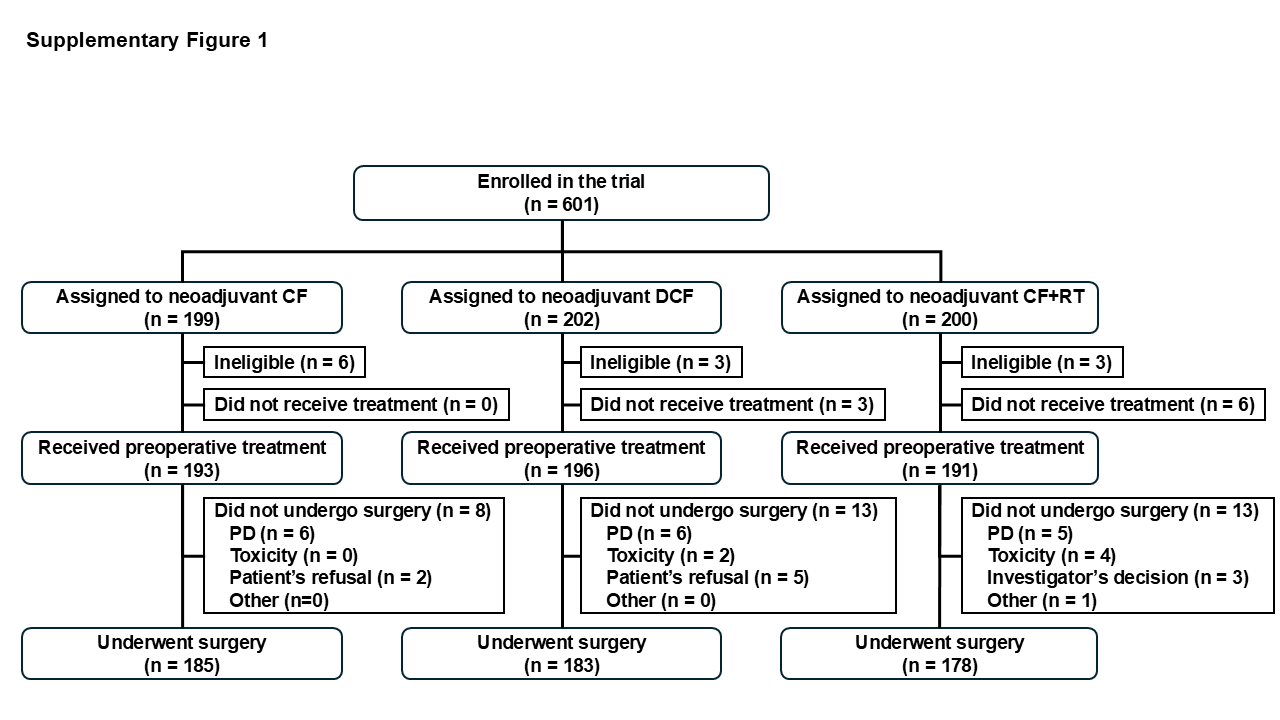


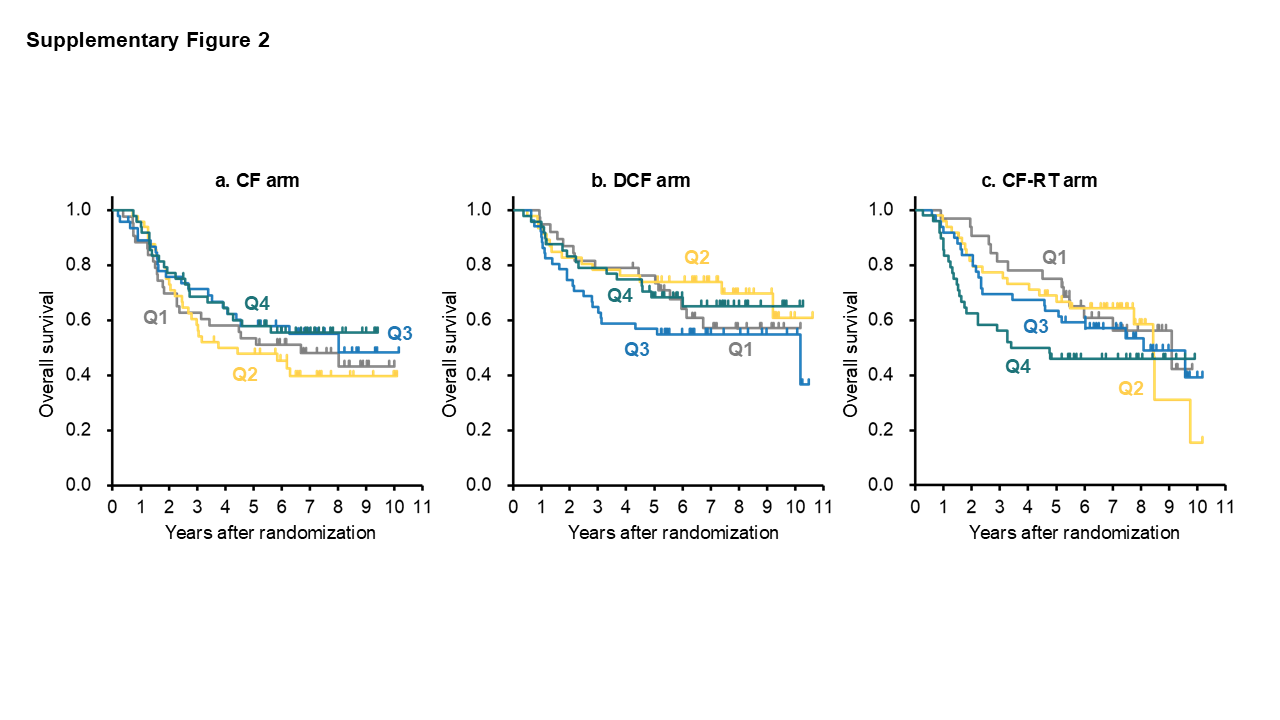


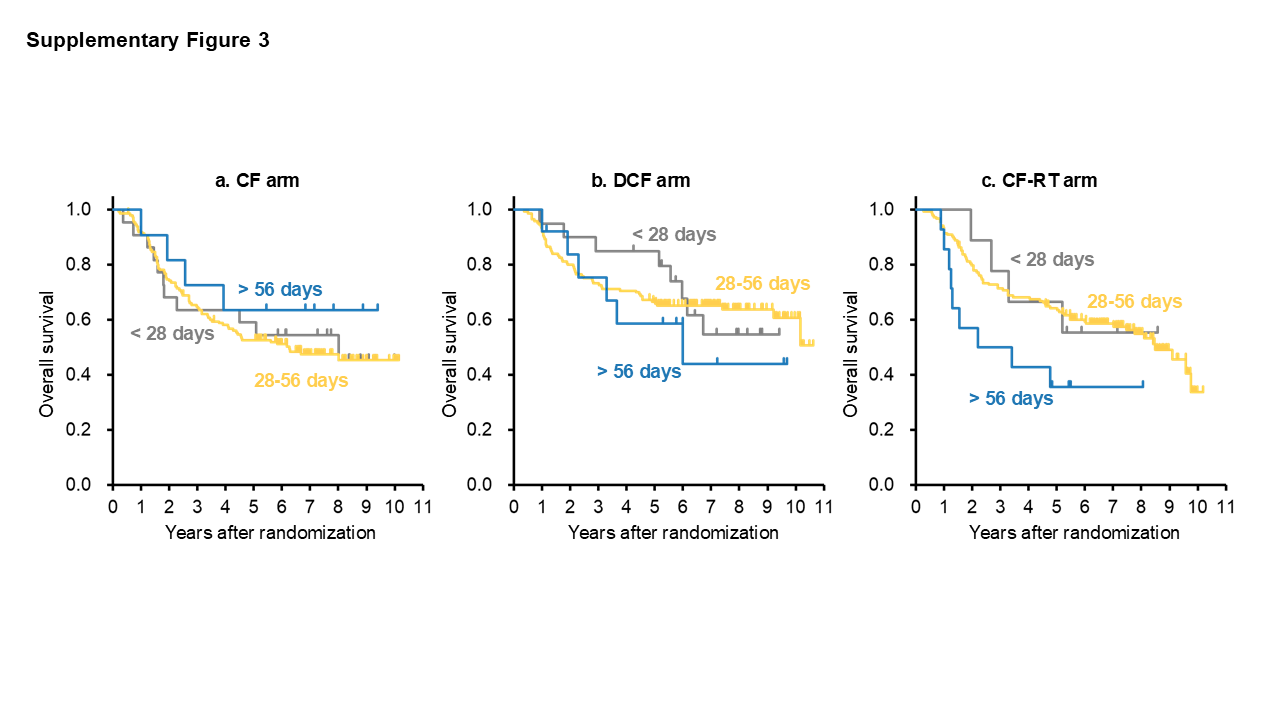


**Supplement Table 1** Univariable and multivariable analysis for progression-free survival

|  |  | CF arm | | | DCF arm | | | CF-RT arm | | |
| --- | --- | --- | --- | --- | --- | --- | --- | --- | --- | --- |
|  |  | Univariable | Multivariable | | Univariable | Multivariable | | Univariable | Multivariable | |
|  |  | HR  (95% CI) | HR  (95% CI) | p-value | HR  (95% CI) | HR  (95% CI) | p-value | HR  (95% CI) | HR  (95% CI) | p-value |
| Age | < 65 | 1 | 1 |  | 1 | 1 |  | 1 | 1 |  |
|  | > 65 | 1.084 (0.743-1.582) | 1.105 (0.745-1.639) | 0.619 | 0.992 (0.643-1.530) | 0.933 (0.587-1.482) | 0.768 | 1.267 (0.840-1.910) | 1.238 (0.814-1.881) | 0.318 |
| Gender | Male | 1 | 1 |  | 1 | 1 |  | 1 | 1 |  |
|  | Female | 0.737 (0.384-1.413) | 0.657 (0.333-1.298) | 0.227 | 0.773 (0.372-1.605) | 0.793 (0.378-1.662) | 0.539 | 0.737 (0.393-1.383) | 0.665 (0.349-1.268) | 0.215 |
| ECOG PS | 0 | 1 | 1 |  | 1 | 1 |  | 1 | 1 |  |
|  | 1 | 0.965 (0.567-1.641) | 0.844 (0.487-1.462) | 0.545 | 0.504 (0.219-1.158) | 0.450 (0.190-1.063) | 0.069 | 0.959 (0.511-1.801) | 0.838 (0.443-1.586) | 0.588 |
| Neoadjuvant  treatment completion | No | 1 | 1 |  | 1 | 1 |  | 1 | 1 |  |
|  | Yes | 0.510 (0.266-0.978) | 0.403 (0.201-0.807) | 0.010 | 1.039 (0.501-2.157) | 1.062 (0.501-2.250) | 0.875 | 0.326 (0.157-0.679) | 0.391 (0.181-0.842) | 0.016 |
| cStage | IB | 1 | 1 |  | 1 | 1 |  | 1 | 1 |  |
|  | II | 0.867 (0.376-1.998) | 0.898 (0.381-2.119) | 0.806 | 4.313 (1.013-18.357) | 4.592 (1.076-19.602) | 0.040 | 0.672 (0.319-1.414) | 0.733 (0.344-1.558) | 0.419 |
|  | III | 1.763 (0.812-3.828) | 1.880 (0.847-4.173) | 0.121 | 5.866 (1.432-24.040) | 6.488 (1.576-26.704) | 0.010 | 1.223 (0.645-2.321) | 1.284 (0.670-2.458) | 0.451 |
| Interval time | Q1 | 1 | 1 |  | 1 | 1 |  | 1 | 1 |  |
|  | Q2 | 1.004 (0.627-1.610) | 1.151 (0.708-1.873) | 0.570 | 0.689 (0.356-1.333) | 0.670 (0.342-1.310) | 0.241 | 1.041 (0.476-2.275) | 1.018 (0.457-2.271) | 0.964 |
|  | Q3 | 0.964 (0.583-1.594) | 1.133 (0.678-1.892) | 0.634 | 0.949 (0.538-1.674) | 0.961 (0.533-1.733) | 0.895 | 0.943 (0.445-1.998) | 0.944 (0.442-2.017) | 0.882 |
|  | Q4 | 0.661 (0.353-1.239) | 0.727 (0.373-1.418) | 0.350 | 0.722 (0.398-1.310) | 0.826 (0.438-1.557) | 0.554 | 1.522 (0.732-3.165) | 1.324 (0.626-2.800) | 0.463 |

**Supplement Table 2** Univariable and multivariable analysis for overall survival stratified by quantile value in each arm

|  |  | CF arm | | | DCF arm | | | CF-RT arm | | |
| --- | --- | --- | --- | --- | --- | --- | --- | --- | --- | --- |
|  |  | Univariable | Multivariable | | Univariable | Multivariable | | Univariable | Multivariable | |
|  |  | HR  (95% CI) | HR  (95% CI) | p-value | HR  (95% CI) | HR  (95% CI) | p-value | HR  (95% CI) | HR  (95% CI) | p-value |
| Age | < 65 | 1 | 1 |  | 1 | 1 |  | 1 | 1 |  |
|  | > 65 | 1.152 (0.766-1.732) | 1.153 (0.754-1.762) | 0.512 | 0.971 (0.605-1.558) | 0.806 (0.492-1.320) | 0.392 | 1.618 (1.042-2.513) | 1.610 (1.026-2.527) | 0.038 |
| Gender | Male | 1 | 1 |  | 1 | 1 |  | 1 | 1 |  |
|  | Female | 0.460 (0.201-1.054) | 0.438 (0.187-1.023) | 0.056 | 0.413 (0.151-1.135) | 0.436 (0.158-1.206) | 0.110 | 0.770 (0.397-1.491) | 0.680 (0.342-1.353) | 0.272 |
| ECOG PS | 0 | 1 | 1 |  | 1 | 1 |  | 1 | 1 |  |
|  | 1 | 1.127 (0.648-1.959) | 0.994 (0.560-1.766) | 0.984 | 0.273 (0.086-0.869) | 0.238 (0.073-0.778) | 0.018 | 0.966 (0.499-1.869) | 0.882 (0.451-1.727) | 0.714 |
| Neoadjuvant  treatment completion | No | 1 | 1 |  | 1 | 1 |  | 1 | 1 |  |
|  | Yes | 0.529 (0.266-1.054) | 0.476 (0.235-0.964) | 0.039 | 0.983 (0.449-2.148) | 0.998 (0.446-2.233) | 0.996 | 0.350 (0.161-0.763) | 0.421 (0.185-0.958) | 0.039 |
| cStage | IB | 1 | 1 |  | 1 | 1 |  | 1 | 1 |  |
|  | II | 0.611 (0.258-1.446) | 0.645 (0.268-1.552) | 0.328 | 7.629 (1.020-57.028) | 8.509 (1.134-63.814) | 0.037 | 0.668 (0.305-1.460) | 0.747 (0.335-1.664) | 0.475 |
|  | III | 1.399 (0.642-3.051) | 1.434 (0.643-3.197) | 0.379 | 9.272 (1.279-67.200) | 11.140 (1.528-81.196) | 0.017 | 1.181 (0.603-2.311) | 1.320 (0.671-2.599) | 0.421 |
| Interval time | Q1 | 1 | 1 |  | 1 | 1 |  | 1 | 1 |  |
|  | Q2 | 1.116 (0.643-1.939) | 1.127 (0.639-1.989) | 0.680 | 0.766 (0.369-1.587) | 0.755 (0.361-1.577) | 0.455 | 1.116 (0.567-2.198) | 0.986 (0.485-2.002) | 0.968 |
|  | Q3 | 0.813 (0.450-1.469) | 1.002 (0.546-1.837) | 0.995 | 1.366 (0.716-2.606) | 1.358 (0.697-2.646) | 0.369 | 1.225 (0.633-2.370) | 1.197 (0.610-2.347) | 0.601 |
|  | Q4 | 0.769 (0.426-1.390) | 0.917 (0.499-1.685) | 0.780 | 0.882 (0.436-1.785) | 1.142 (0.544-2.398) | 0.726 | 1.759 (0.918-3.373) | 1.505 (0.767-2.952) | 0.235 |

**Supplement Table 3** Univariable and multivariable analysis for overall survival stratified by absolute value

|  |  | CF arm | | | DCF arm | | | CF-RT arm | | |
| --- | --- | --- | --- | --- | --- | --- | --- | --- | --- | --- |
|  |  | Univariable | Multivariable | | Univariable | Multivariable | | Univariable | Multivariable | |
|  |  | HR  (95% CI) | HR  (95% CI) | p-value | HR  (95% CI) | HR  (95% CI) | p-value | HR  (95% CI) | HR  (95% CI) | p-value |
| Age | < 65 | 1 | 1 |  | 1 | 1 |  | 1 | 1 |  |
|  | > 65 | 1.152 (0.766-1.732) | 1.164 (0.763-1.775) | 0.480 | 0.971 (0.605-1.558) | 0.835 (0.515-1.352) | 0.463 | 1.618 (1.042-2.513) | 1.612 (1.031-2.522) | 0.036 |
| Gender | Male | 1 | 1 |  | 1 | 1 |  | 1 | 1 |  |
|  | Female | 0.460 (0.201-1.054) | 0.406 (0.174-0.947) | 0.037 | 0.413 (0.151-1.135) | 0.415 (0.150-1.150) | 0.091 | 0.770 (0.397-1.491) | 0.637 (0.322-1.262) | 0.196 |
| ECOG PS | 0 | 1 | 1 |  | 1 | 1 |  | 1 | 1 |  |
|  | 1 | 1.127 (0.648-1.959) | 1.025 (0.575-1.828) | 0.934 | 0.273 (0.086-0.869) | 0.236 (0.074-0.758) | 0.015 | 0.966 (0.499-1.869) | 0.887 (0.456-1.728) | 0.725 |
| Neoadjuvant  treatment completion | No | 1 | 1 |  | 1 | 1 |  | 1 | 1 |  |
|  | Yes | 0.529 (0.266-1.054) | 0.459 (0.226-0.932) | 0.031 | 0.983 (0.449-2.148) | 1.089 (0.485-2.444) | 0.836 | 0.350 (0.161-0.763) | 0.394 (0.175-0.889) | 0.025 |
| cStage | IB | 1 | 1 |  | 1 | 1 |  | 1 | 1 |  |
|  | II | 0.611 (0.258-1.446) | 0.654 (0.272-1.572) | 0.343 | 7.629 (1.020-57.028) | 7.671 (1.020-57.707) | 0.048 | 0.668 (0.305-1.460) | 0.719 (0.326-1.584) | 0.413 |
|  | III | 1.399 (0.642-3.051) | 1.461 (0.657-3.247) | 0.353 | 9.272 (1.279-67.200) | 10.392 (1.429-75.561) | 0.021 | 1.181 (0.603-2.311) | 1.253 (0.637-2.466) | 0.514 |
| Interval time | Early | 1 | 1 |  | 1 | 1 |  | 1 | 1 |  |
|  | Middle | 1.051 (0.559-1.977) | 1.199 (0.623-2.306) | 0.586 | 0.996 (0.474-2.093) | 1.130 (0.531-2.406) | 0.751 | 1.051 (0.383-2.883) | 1.001 (0.362-2.772) | 0.998 |
|  | Late | 0.652 (0.207-2.047) | 0.737 (0.230-2.360) | 0.607 | 1.366 (0.474-3.942) | 1.425 (0.477-4.261) | 0.526 | 2.275 (0.699-7.400) | 1.987 (0.603-6.544) | 0.259 |
